# Inconsistencies in care: a UK and Ireland survey exploring acute vestibular service provision in adult major trauma centres

**DOI:** 10.3389/fneur.2026.1829958

**Published:** 2026-05-25

**Authors:** Rebecca M. Smith, Vassilios Tahtis, Abby Newdick, Claire Harris, Luke Wilkinson, Catriona Ralph, Alec Roberts, Jeremy Corcoran

**Affiliations:** 1 Centre for Vestibular Neurology, Imperial College London, London, United Kingdom; 2 King’s College Healthcare NHS Foundation Trust, London, United Kingdom; 3 St George’s Hospital NHS Foundation Trust, London, United Kingdom; 4 Salford Royal Hospital NHS Foundation Trust, Salford, United Kingdom; 5 Imperial College Healthcare NHS Trust, London, United Kingdom; 6 Queen Elizabeth University Hospital, Glasgow, United Kingdom; 7 Bolton NHS Foundation Trust, Bolton, United Kingdom; 8 Guy’s and St Thomas’ NHS Foundation Trust, London, United Kingdom

**Keywords:** rehabilitation, service provision, survey, traumatic brain injury, vestibular dysfunction

## Abstract

**Background:**

Vestibular dysfunction is common following traumatic brain injury (TBI). Previous research has noted patients are not routinely assessed or treated acutely, due to uncertainty around healthcare professionals’ roles and their limited knowledge and skills. This research however was limited to two UK London major trauma centres (MTCs). The present study aimed to explore current provision for acute post-traumatic vestibular assessment and treatment in UK and Ireland MTCs.

**Methods:**

An online survey was developed and refined with input from healthcare professionals. The final survey was distributed between April and August 2024 to healthcare professionals working in 32 adult MTCs in England, Wales, Scotland, and Ireland. Data were analysed using Excel.

**Results:**

Surveys were completed at 28/32 (87.5%) MTCs. 13/28 (38%) of respondents indicated routine vestibular assessment and treatment were provided. Barriers to routine assessment and treatment included knowledge and skills deficiencies and the absence of protocols. Geographic variation was evident with 75% of centres in London and Scotland reporting higher rates of routine vestibular assessment and treatment compared to 20%–25% of centres in other regions.

**Conclusion:**

Our findings show adult acute vestibular service provision is inconsistent across MTCs in the UK and Ireland, with the potential to cause inequalities in health and care outcomes. Strategies to address inconsistent service provision include training and the development of pathways and guidelines both nationally and locally.

## Introduction

Vestibular dysfunction following traumatic brain injury (TBI) is caused by injury to peripheral (i.e., inner ear and nerve) or central (i.e., brain) vestibular structures and can result in dizziness or imbalance (1). These symptoms are common, affecting up to 80% of ambulant moderate-to-severe acute (in-patients within a major trauma centre) patients with TBI. In this manuscript, we refer to the Malec et al. classification (2) for the definition and severity of TBI (3, 4) and focus on moderate–severe TBI as this is predominantly where the evidence exists. Prevalent post-traumatic vestibular symptoms include those arising from peripheral dysfunction (e.g., benign paroxysmal positional vertigo [BPPV] and acute peripheral unilateral vestibular loss) and those of central origin (e.g., centrally mediated gait ataxia and migraine phenotype headache) (5). Patients may present with multiple vestibular diagnoses (5, 6) and with absent vestibular perception (7, 8), increasing the complexity of management and the risk of missed diagnoses.

Early or acute management of vestibular dysfunction following TBI appears to be feasible (9) and important from a patient perspective (10). Indeed, research indicates the absence of a diagnosis or delays to diagnosis and treatment acutely can adversely impact patients’ physical and psychosocial outcomes and quality of life (11–13). Patients who do not receive early diagnosis and management often attend primary care for onward referral to specialists. This pathway is associated with delays of up to 93 weeks from first referral to treatment for patients with BPPV (14), high healthcare costs due to unnecessary imaging or tests (15), increased risk of falls due to associated imbalance (16), reduced quality of life, and the risk of developing maladaptive coping strategies and chronic symptoms such as in persistent postural perceptual dizziness (17). In turn, chronicity of vestibular symptoms (symptoms lasting longer than three months) is associated with poorer quality of life, higher levels of disability, delayed return to work and elevated anxiety and depression (17–19). Heightened falls risk and falls in patients with TBI are particularly relevant as recent research indicated unintentional trauma (i.e., falls) was associated with increased mortality in TBI survivors (20). Falls also result in significant physical, psychological and healthcare costs (21–23). It follows that efforts to address modifiable risk factors to prevent falls, such as vestibular dysfunction, must be made as per recent national priorities (24, 25).

Despite the need for early intervention, specific clinical recommendations for acute vestibular assessment or treatment do not feature in UK clinical guidelines on early management of traumatic brain injury (26). Research has also indicated vestibular assessment and treatment during acute TBI are not commonplace, with considerable variation in practice observed across neighbouring major trauma centres (27). This qualitative study identified barriers to routine management of vestibular dysfunction including role uncertainty among healthcare professionals, and knowledge and skill-based barriers (25). Such data pertaining to service provision for patients with post-traumatic vestibular dysfunction were limited to two major trauma centres in London. There are currently no data relating to service provision in other regions, and thus it is unclear whether clinical practice differences between major trauma centres (MTCs) are more wide spread. Regional disparities in the availability or quality of services could mean patient care is influenced more by geographical location than individual clinical need or best practice. The aim of this study was therefore to explore current clinical service provision for adults with post-traumatic vestibular dysfunction attending MTCs across UK and Ireland.

## Methods

### Study design and ethics

This study was a cross-sectional survey administered online using QualtricsXM (Qualtrics, Provo, UT). The survey was targeted at practising healthcare professionals managing traumatic brain injury patients at adult MTCs in the UK and Ireland. It was sent to all 32 MTCs; 24 in England, four in Scotland, one in Wales and three across Northern Ireland and the Republic of Ireland. MTCs specialising only in paediatrics were excluded. The survey link was distributed via email by the lead author (RMS) to designated trauma therapy team leads, rehabilitation co-ordinators or a suitable alternative identified during a phone call by RMS. The survey opened on the 20/04/2024 and closed on 04/09/2024. Consent to participate in the survey was implied by completion and online submission of the survey. In the pre-survey text, participants were informed their participation was voluntary and results would be anonymous. The lead author sent one reminder 2 weeks after the initial invitation if no response had been forthcoming. Respondents were able to complete the survey in more than one session. This study was a service evaluation and as per the Health Research Authority decision tool did not require ethical approval. The study was conducted in accordance with the Declaration of Helsinki (2024).

### Survey development

The survey was developed by the lead author (RMS) in conjunction with input from five Physiotherapists and Occupational therapists from UK MTCs. The theoretical domains framework (TDF) (28) and the Consolidated Framework for Implementation Research (CFIR) (29) were used to guide decisions about broader content. The initial survey was piloted by five trauma ward therapists and subsequently refined following feedback. Cognitive interviewing was also conducted to ensure the validity of the survey. This is a qualitative method to explore how the target population interpret and respond to items or questions (30). Two experienced trauma ward therapists participated in a Cognitive interview with the lead author (RMS). An intensive interviewing paradigm (30) was used, in which the therapists completed the survey whilst providing their interpretation of the question and elaborating on their answers. Feedback was used to revise or reword individual items (i.e., to improve readability). The final draft was reviewed by clinical experts in the field. The survey was available in English and had 40 questions. The survey used adaptive questioning (items displayed depending on responses to previous items) to reduce the number and complexity of questions. There were a maximum of three questions per page. Key questions mandated a ‘forced’ response; that is, respondents could not move through the survey until they had answered the question. The survey had five sections (Table 1). Most questions were closed in nature, yielding either interval (i.e., a rating of confidence in assessing posterior canal BPPV) or ordinal data (e.g., yes or no response). An open question was posed at the end of the survey requesting participants to add any further details of relevance. A copy of the survey is provided in Supplementary Material S1. The survey took approximately 15 min to complete. No incentives were offered for survey completion. Findings are reported in accordance with the Checklist for Reporting Results of Internet ESurveys (CHERRIES) (31).

**Table 1:** Sections of the survey.

Section	Description of items
I—Demographics (Questions 1–3)	Questions about the MTC respondents were based at, their profession and how many years they had worked in trauma.
II—Knowledge and training (Questions 4–5)	The range of post-traumatic vestibular disorders respondents were aware of, and the amount and type of training they had been exposed to.
III—Routine assessment and treatment (Questions 6–9)	Whether patients were routinely assessed for vestibular dysfunction or, if not whether assessment was guided by the presence of subjective complaints (i.e., dizziness) and/or objective signs (i.e., nystagmus). Items also intended to find out which healthcare professional/s were responsible for managing patients with vestibular dysfunction, if there was a protocol or pathway in place and what tests were included in a vestibular assessment.
IV—BPPV (Questions 10–27)	Assessment and treatment of posterior and horizontal canal BPPV. Respondents were asked to specify which and how many healthcare professionals per MTC were able to assess and/or treat BPPV. Data were also gathered on perceptions of assessing and treating BPPV as a priority in this cohort, overall confidence in assessment and treatment, confidence in more specific aspects of assessment (e.g., interpreting eye movements, identifying the affected side), and whether patients were referred on.
V—Peripheral vestibular hypofunction and changes required to services (Questions 28–40)	Whether their teams were able to diagnose a peripheral vestibular hypofunction, how that diagnosis came about, whether patients were treated acutely and if they made onward referrals. Finally, respondents were asked whether they perceived changes were needed to how trauma centres managed vestibular dysfunction and what sort of changes were required.

### Data analysis

Data were analysed descriptively. Normally and non-normally distributed data are reported as means (SDs) and medians (IQRs), respectively. Fisher’s exact test was used to determine whether there was an association between categorical data relating to the presence of a pathway or protocol and the routine nature of vestibular assessment and treatment. For both variables, only yes and no responses were included. Following these analyses of data from the whole sample, centres were grouped into geographic areas to explore any regional differences in practice. Differences between these data sets were assessed using descriptive statistics only as the small number of results in certain sub-categories precluded formal statistical testing. London was incorporated as four MTCs (four responses), north England as nine centres (10 responses), the Midlands as four centres (five responses), South England as five centres (eight responses), Scotland as four centres (five responses), Wales as one centre (one response) and Republic of Ireland as one centre (one response). Six centres had more than one response (Supplementary Material S2). In these centres, an unanimity rule was applied, that is responses were required to be consistent in order for practice to be confirmed. In four of the centres, responses were conflicting, that is, reported practice varied and was therefore marked as ‘unclear’. Free text responses were analysed using thematic analysis (32). RMS coded the data on a line by line basis. Codes were subsequently reduced, grouped, and abstracted into themes. Themes were reviewed and refined with the research team. Data were analysed using Excel (version 2021; Microsoft Corporation, Redmond, WA, United States).

## Results

28 of 32 centres (87.5%) responded to the survey with 34 responses received in total. A single response was received from 22 MTCs, while the remaining six MTCs contributed two responses each, typically from a physiotherapist and occupational therapist (see Supplementary Material S2); these duplicate responses were submitted unintentionally rather than by design. All 34 responses were analysed. As adaptive questioning was employed throughout the survey, item level denominators may differ depending on participants’ prior responses.

### Demographics

Surveys were completed by physiotherapists, occupational therapists, nurses, a trauma therapy coordinator or a physiotherapist and occupational therapist jointly (Table 2). Healthcare professionals on average had worked in trauma for 7 years (range between 0–20 years).

**Table 2:** Respondents’ professions and experience of trauma work.

Healthcare profession (number of responses/34, %)	Median years worked in trauma (IQR)
Physiotherapist (28/34, 82%)	6 (4.5–3.75)
Occupational therapist (2/34, 6%)	5.5 (6.75–4.25)
Nurse (2/34, 6%)	8.5 (10.25–6.75)
Trauma therapy coordinator (1/34, 3%)	11
Physiotherapist and occupational therapist (1/34, 3%)	2

### Knowledge and training

Awareness of BPPV as a post-traumatic vestibular diagnosis was high (32/34, 94% of respondents), but awareness of other diagnoses less so (post-traumatic vestibular hypofunction 19/34, 56%; vestibular migraine 17/34, 50%). 24/34 (71%) of respondents reported one or more members of their team had either received in-house training or attended external courses (23/34; 67%) of relevance, whilst fewer had worked in vestibular rehabilitation (6/34, 19%) or had completed a masters level qualification or above (2/31, 6%) in the field of neuro-otology.

### Routine assessment and treatment

**Figure 1:**
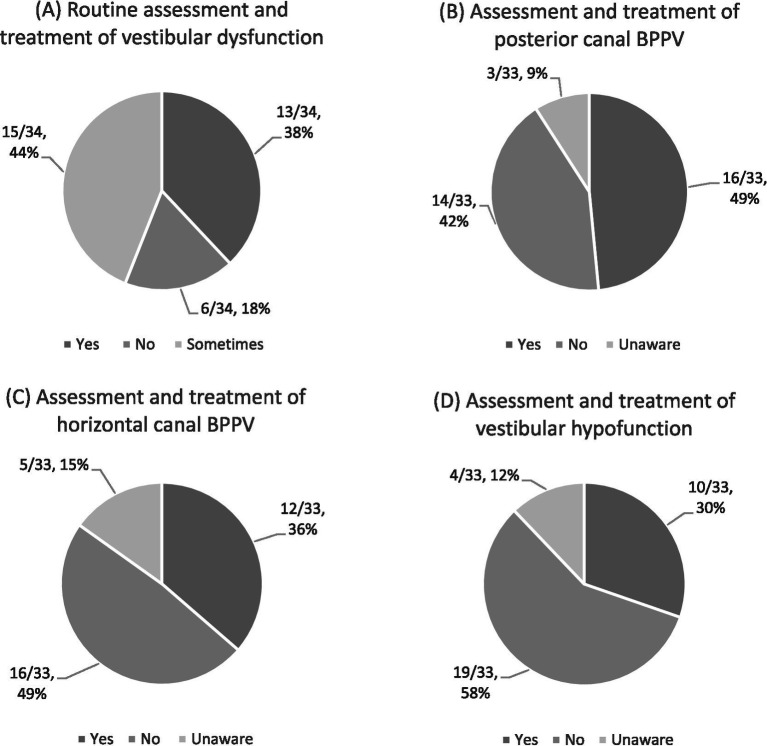
**(A)** Routine assessment and treatment of vestibular dysfunction. **(B)** Assessment and treatment of posterior canal BPPV **(C)** Assessment and treatment of horizontal canal BPPV. **(D)** Assessment and treatment of vestibular hypofunction.

Survey responses indicated that assessment and treatment varied in the frequency of routine completion (Figure 1A). Respondents who indicated that assessment and treatment were completed “sometimes” (15/34; 44%), were asked whether this was guided by the presence of subjective reports of dizziness (13/15, 87%), objective signs such as nystagmus (12/15, 80%) or specific features related to the injury (5/15, 33%). Of all respondents, 56% (15/27) reported assessment was only indicated if patients reported subjective dizziness or imbalance.

**Figure 2:**
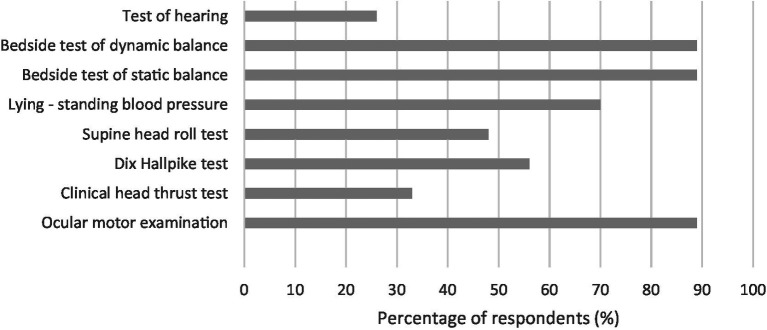
Clinical components of a vestibular assessment.

21 respondents reported barriers to routine vestibular assessment and treatment. All respondents (21/21,100%) identified healthcare professionals’ knowledge and skills were a barrier, while 19/21 (90%) reported a lack of protocols and pathways and 11/21 (52%) identified limited time or capacity as barriers. 26/34 (79%) respondents indicated that their hospital trust did not have a pathway or protocol for managing patients with vestibular dysfunction. The presence of a pathway or protocol was significantly associated with increased routine assessment and treatment (OR = 9.43, *p = 0*.026). Trauma ward physiotherapists were most commonly responsible for post-traumatic vestibular assessment and treatment (20/28; 71%), however variation was evident amongst centres with doctors, specialist nurses, occupational therapists and visiting specialists also involved. Common components of a routine vestibular assessment (reported by *n* = 27) are shown in Figure 2.

### BPPV assessment and treatment

**Figure 3:**
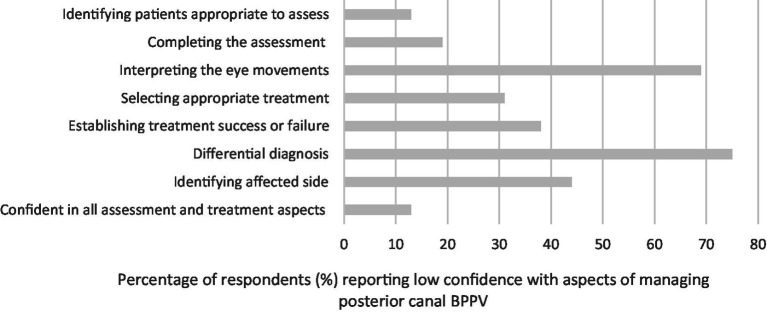
Confidence rates relating to aspects of management of posterior canal BPPV.

Assessment and treatment of posterior and horizontal canal BPPV were inconsistently performed across major trauma centres (Figures 1B,C). On average, BPPV assessment and treatment in general was considered by 20 respondents to be a medium-high priority in acute TBI (rated as 57/100). Respondents were of the opinion that physiotherapists had more capacity to undertake BPPV assessment and treatment than occupational therapists, nurses, and doctors (Supplementary Material S3). The most common barriers to assessing and treating BPPV were knowledge and skills (17/30; 57%), time or staff capacity (7/30; 23%) and apprehension about completing procedures (5/30; 17%). Respondents were more confident with posterior canal BPPV assessment (median confidence 62.5%; IQR 48.25–81.75) and treatment (median confidence 53.5%; IQR 38.25–70.5 than with horizontal canal BPPV assessment and treatment (50.5%; IQR 37.5–60.25 and 48.5%; IQR 37.5–52% respectively). Respondents noted specific aspects of assessment and treatment (e.g., interpreting eye movements and differential diagnosis) were associated with reduced confidence (Figure 3).

Respondents most commonly indicated posterior canal BPPV would be treated with an Epley or Semont manoeuvre (24/33; 73%). Advice and Brandt-Daroff exercises were less frequently used (6/33; 18% and 2/33; 6% respectively). Treatment options provided for horizontal canal BPPV included log roll manouevre (10/23, 43%), Gufoni manoeuvre (7/23, 30%), advice (4/23, 17%), and forced prolonged positioning (2/23, 9%). When asked about onward referrals for patients with BPPV, 13/33 (29%) would always refer, 6/33 (18%) would never refer and 14/33 (42%) would sometimes refer. Respondents reported that a lack of community or outpatient services, clearly defined clinical indicators, pathways, or protocols were reasons for not referring patients routinely. Referrals were most frequently made following incomplete treatment (19/54; 35%), for patients with suspected BPPV (i.e., not formally diagnosed—18/54; 33%) and for patients who had been diagnosed but not treated (15/54; 28%). Referrals were directed to eight different services including general practice, audiology, TBI outpatients, and outpatient neuro therapy and community rehabilitation.

### Peripheral vestibular hypofunction

Responses relating to assessment and treatment of vestibular hypofunction are shown in Figure 1D. Frequent barriers to diagnosing vestibular hypofunction included insufficient skills or knowledge (21/23; 91%), lack of practical skills (17/23; 74%), lack of access to vestibular function testing (17/23; 74%) and lack of senior or consultant level support (12/23; 52%). Of those able to diagnose vestibular hypofunction, that diagnosis was reached if there was an abnormal clinical head trust test (7/17; 41%), a peripheral vestibular nystagmus (6/17; 35%), and/or an indicative finding on vestibular function testing (3/17; 18%). Treatment for vestibular hypofunction was provided in 8/14 (57%) MTCs and included gaze stability exercises, static and dynamic balance exercises, and advice not to avoid normal head movements. 8/31 (26%) and 10/31 (32%) of respondents, respectively, reported always and sometimes referring patients with a diagnosed vestibular hypofunction on to specific services. Referrals were most commonly directed to outpatient vestibular therapy services (41%; 11/27) although respondents reported a range of other referral destinations. The most frequent barrier to making onward referrals was the lack of available outpatient or community services (18/29; 62%).

### Changes to vestibular service provision

**Figure 4:**
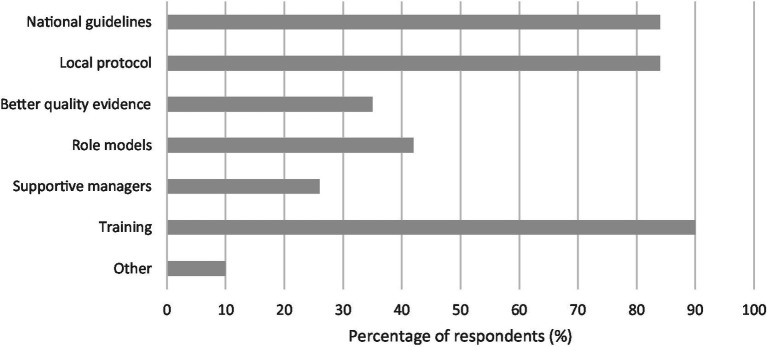
Respondents’ solutions for improved management of vestibular dysfunction in Major Trauma.

97% (31/32) of respondents reported that changes were necessary to the way centres manage vestibular dysfunction. Solutions put forward by respondents to mediate change are shown in Figure 4.

### Geographical variation and within Centre variation

**Figure 5:**
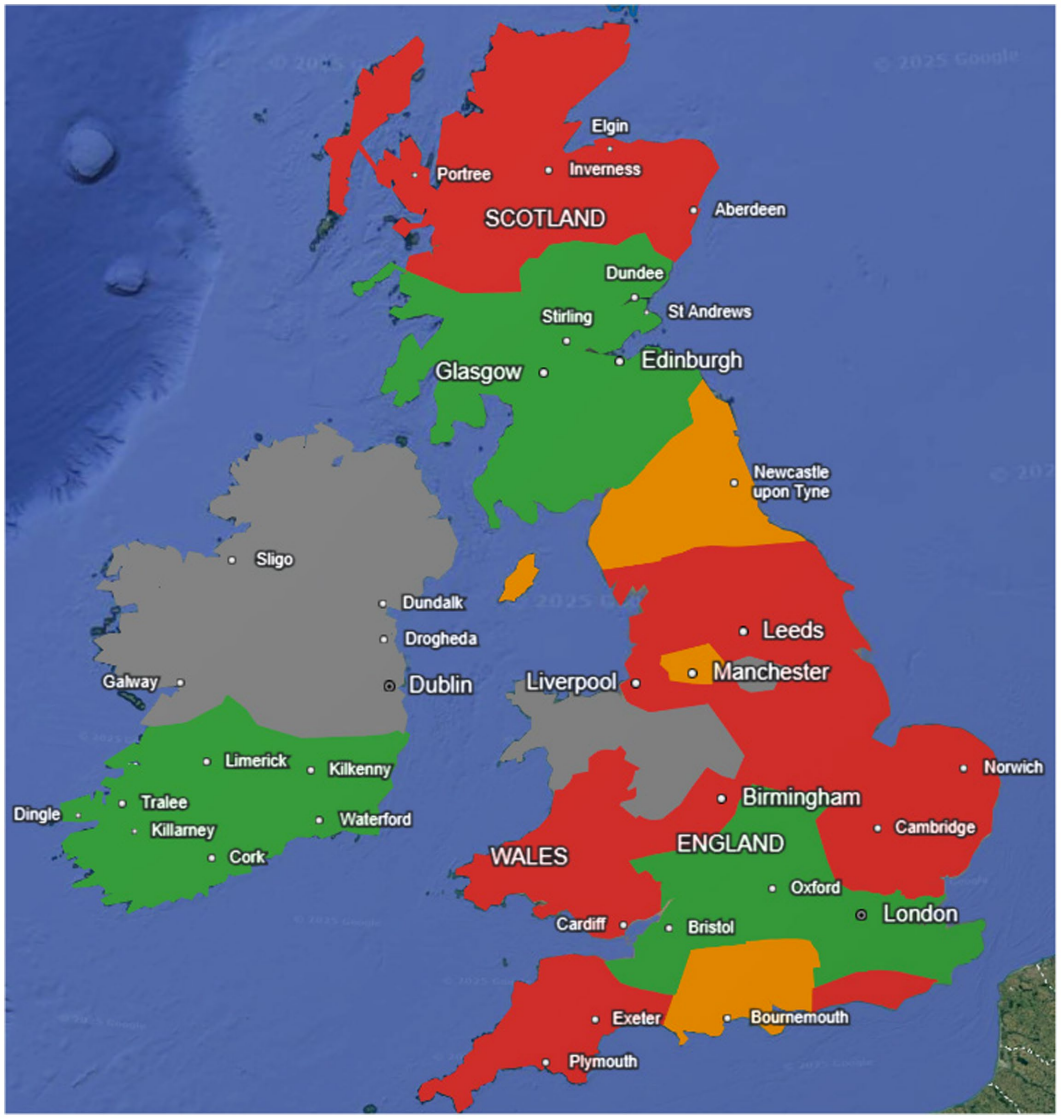
Geographical variation in routine assessment and treatment of posterior canal BPPV. Shaded red areas denote areas of no routine assessment or treatment. Shaded orange areas denote areas in which centres reported differing practices. Green shaded areas denote posterior canal BPPV is assessed and treated. Areas shaded grey denote areas where no response was recorded. The map was created using the trauma networks map. Boundaries are approximate. https://www.google.com/maps/d/u/0/viewer?mid=1bJvF8AG4F0RjF7wVjJicsnKC3qOu-TT6&ll=54.86967561975241%2C-0.7759855546152492&z=5.

Large geographical variation was noted in centres’ responses about whether post-traumatic vestibular dysfunction was routinely assessed or treated (Table 3). More specifically, centres in London, Scotland and the Republic of Ireland reported higher rates of routine assessment and treatment of posterior canal BPPV (Figure 5). Confidence in BPPV assessment and treatment also varied. Notably, few centres outside London or Scotland had pathways or protocols in place for managing post-traumatic vestibular dysfunction.

**Table 3:** Geographic variation in aspects of vestibular service provision.

Region (Number of responses)	Routine assessment and treatment	Pathway in place	Confidence in PC assessment and treatment	View of BPPV as a priority	Managing hypofunction
London (4)	75%	50%	70%	92%	50%
North England (10)	20%	10%	32%	44%	10%
Midlands (5)	25%	0%	73%	66%	0%
South England (8)	40%	20%	40%	48%	40%
Scotland (5)	75%	50%	73%	64%	75%
Wales (1)	0%	0%	N/A	100%	0%
Ireland (1)	0%	0%	39%	38%	0%

PC, posterior canal. Centres in London—St Mary’s Hospital, Royal London Hospital, St George’s Hospital and King’s College London. Centres in North England—Royal Victoria Infirmary, James Cook University Hospital, Aintree University Hospital*, Royal Preston Hospital, Salford Royal Hospital, Manchester Royal Infirmary, Leeds General Infirmary* and Hull University Teaching Hospital. Centres in the Midlands—John Radcliffe Hospital, Queens Medical Centre Nottingham* University Hospitals Coventry and Warwickshire, Queen Elizabeth Hospital Birmingham. Centres in South and East England were Southmead Hospital*, Addenbrookes Hospital*, Royal Sussex County Hospital, Southampton General Hospital*, Derriford Hospital Plymouth. Centres in Scotland—Royal Infirmary Edinburgh*, Queen Elizabeth University Hospital Glasgow, Aberdeen Royal Infirmary, Ninewells Hospital Dundee. Centres in Wales and Republic of Ireland—Cardiff Royal Infirmary and Cork University Hospital. * Denotes centres with two responses.

### Qualitative analysis of textual data

Thematic analysis was applied to free text comments entered against the ‘other’ response option questions (*n* = 5 items), as well as to comments made in relation to the last item which permitted respondents to add views which had not been expressed previously (*n* = 9 responses). Analysis generated three main themes: (1) Training and education, (2) Provision of resources and (3) De-prioritisation of dizziness in acute TBI.

Training and education—Respondents suggested training and education was lacking in vestibular assessment and treatment, and should be part of both undergraduate curricula and post-registration training. This was felt to be important for clinical capacity, continuity of services and succession planning.

*“Lots more training required! We had one vestibular specialist who doesn’t practice now, and her service was decommissioned”* Physiotherapist, Nottingham

*“The provision of basic education around basic vestibular anatomy and identification of impairments on university curriculums”* Physiotherapist, Salford

Provision of resources and evidence – Respondents noted that a lack of resources, staff capacity and evidence were barriers to routine assessment and treatment. Protocols, case studies and videos were suggested as means to improving confidence in vestibular assessment and treatment.

*“Assessment protocol (and advice on how to deal with any complications) would give staff more confidence”* Physiotherapist, Sussex

*“The challenges I have are a lack of vestibular service (outpatients) within my trauma network”* Specialist Nurse, Thames Valley

*“Evidence in relation to assessment and treatment specific to post TBI; WHEN to assess and treat post TBI can be challenging”* Physiotherapist, Scotland

De-prioritisation of dizziness acutely—Respondents perceived dizziness was not always regarded as a clinical priority, due to competing post-TBI sequalae or its inconspicuous nature, as reflected by how infrequently it was routinely managed and followed up.

*“I think dizziness is downplayed when compared to cognitive deficits in TBI screens on the ward. Patients are rarely followed up by Neurologists in minor head injury if they present with dizziness on the ward but would be if they had any cognitive deficits”* Physiotherapist, London

*“Patients are sometimes independent and therefore a low priority for physiotherapist review”* Physiotherapist, Cambridge

## Discussion

The aim of this study was to explore service provision for adults with acute post-traumatic vestibular dysfunction in the UK and Ireland. The objective was to determine assessment and treatment practices, knowledge and skill levels, and barriers to optimal care. The findings highlight significant variation in clinical practices, familiarity with vestibular dysfunction, and resources available to healthcare professionals, plus key areas for service improvement.

### Variation in clinical practice and geographic differences

One of the key findings of this study was the variation in how vestibular dysfunction, particularly BPPV, was assessed and treated across major trauma centres. While 49% of respondents reported there being routine assessment and treatment of posterior canal BPPV at their centre, only 36% reported similar practices for horizontal canal BPPV. This discrepancy could be attributed to several factors, including clinician confidence, availability of resources, differing priorities within trauma teams and the lack of consensus guidelines available. Further, the majority of respondents (56%) reported assessments were only carried out if patients reported symptoms (e.g., dizziness or imbalance). This is problematic, since vestibular agnosia, an obtunded perception of dizziness, affects approximately a third of patients with moderate–severe TBI (7) and is noted to negatively impact clinicians’ ability to identify vestibular diagnoses (such as BPPV). We therefore recommend that patients are not assessed based on symptom report alone.

It is notable that trauma ward physiotherapists were primarily responsible for post-traumatic vestibular assessment and treatment, although this was not universal across the centres. Prior research notes that patients often have multiple vestibular diagnoses, with complex presentations (6), which often necessitate multidisciplinary working and discussion. National (UK) Trauma Rehabilitation guidelines (33) for Physiotherapists working in TBI do include vestibular assessment and treatment, however there is a clear need for more detailed and locally-tailored guidance on how to adopt the recommendations. Of note, National (UK) rehabilitation guidelines for Occupational therapists (33) working in TBI include assessment of dizziness, and BPPV, but not their treatment. Previous studies have found Physiotherapists *and* Occupational therapists are well placed to assess and treat conditions such as BPPV (27) and, following appropriate training are able to undertake assessment and treatment with confidence and accuracy (9, 34). Future research should be aimed at developing condition-specific, rather than profession-specific, guidance for this cohort, something which is currently lacking.

Variation in practice was also apparent on a geographic dimension, with centres in London, Scotland, and (the Republic of) Ireland more likely to report routine assessment and treatment of BPPV and greater confidence in performing these procedures, compared to centres in North England, the Midlands, and Wales. This geographic variation highlights the influence of local healthcare structures and access to specialized services, and that there may be regional disparities in medical and therapy training, and in resource allocation. A recent feasibility trial across three London major trauma centres involved training trauma ward therapists to assess and treat post-traumatic BPPV (9), and may have influenced practice within this region. The high concentration of multidisciplinary balance clinics in London (including at St George’s Hospital, Guys’ Hospital and University College London Hospital) may also have some bearing on the higher levels of skills and knowledge found by the present study, although it is acknowledged that little data exists on balance clinics in other areas of the UK and Ireland. Practice variation was also recorded within individual centres. Duplicate responses from four centres showed inconsistencies, perhaps indicating either heterogenous practice, differing interpretation of the questions or incomplete awareness of the practice in question.

### Knowledge and training gaps

A further key finding from this survey is the disparity in knowledge across MTCs regarding different types of post-traumatic vestibular dysfunction. While awareness of benign paroxysmal positional vertigo (BPPV) as a post-traumatic vestibular diagnosis was high (94%), less than 60% of respondents were aware of other conditions such as vestibular hypofunction (56%) or vestibular migraine (50%). This suggests that conditions, which are still relatively prevalent yet potentially more debilitating than BPPV, may not be as well understood. The reasons for this are unclear, however may relate to a lack of evidence regarding prevalence, diagnosis, and treatment in this cohort. Notably, of those respondents who were able to assess vestibular hypofunction, this was diagnosed using appropriate clinical and laboratory tests (e.g., video head impulse testing). This in line with guidance which recommends video head impulse testing as a first line laboratory test for detecting a vestibular hypofunction (35). Respondents reported managing vestibular hypofunction using rehabilitation techniques (e.g., gaze stability exercises and balance and gait training) which are recommended in guidelines for patients with vestibular hypofunction (36), albeit the survey did not extend to probing respondents about the dose of exercises, limiting the comparison.

Moreover, despite a relatively high percentage (71%) of respondents reporting having had some form of training, the majority had not participated in specialised vestibular rehabilitation training, and only a small percentage (6%) had achieved advanced qualifications in the field. These findings are in agreement with previous research (27), indicating a significant gap in expertise and role models and highlighting the need for better targeted training at both undergraduate and post-graduate levels to equip healthcare professionals with the necessary skills to manage a range of vestibular disorders. These data are echoed in other work evaluating therapist-led management of vestibular conditions within (37) and outside the context of traumatic brain injury (38–40). Missed or delayed diagnoses are potential consequences of training and knowledge gaps (7) which have important downstream impacts for patients.

### Barriers to effective assessment and treatment

Several barriers to effective assessment and treatment were identified, with the most prominent being a lack of knowledge and skills, the absence of standardised protocols or pathways, and insufficient time or staff capacity. Notably, 79% of respondents indicated that their hospital trust lacked a formal pathway or protocol for managing patients with vestibular dysfunction, which was significantly associated with a lack of routine assessment and treatment. Other evidence in traumatic brain injury (41) and more general clinical care (42) point to improvements in quality of care and reductions in length of stay following implementation of clinical pathways.

### Confidence in BPPV assessment and treatment

Although physiotherapists were identified as having the greatest capacity to assess and treat BPPV, there was a marked difference in confidence levels across the two main types of BPPV seen in acute TBI. Respondents were generally more confident in managing posterior canal BPPV compared to horizontal canal BPPV. This could be due to the relatively low prevalence of horizontal canal BPPV, meaning dedicated assessment and treatment procedures are less commonly practiced or taught. Furthermore, apprehension regarding performing unfamiliar procedures, combined with the lack of formal training, may explain why some practitioners expressed reduced confidence in managing cases of horizontal canal BPPV. Prior in-depth qualitative work (27) has provided some insight into the underpinning factors why physiotherapists, as opposed to other healthcare professionals, are more likely to assess and treat BPPV, including role and training based barriers. It was notable that respondents expressed reduced confidence in differential diagnosis and interpreting eye movements, indicating a need for further specific training.

### Referral practices and barriers

The survey revealed inconsistent referral practices for BPPV and vestibular hypofunction, with a large proportion of respondents either rarely referring patients or only referring under specific circumstances. A lack of community or outpatient services was frequently cited as a barrier to onward referrals. This highlights a critical gap in the continuity of care for patients with vestibular dysfunction following trauma, particularly for those who require ongoing vestibular rehabilitation. While some patients are referred to outpatient vestibular therapy services, others may not have access to these services, further exacerbating the challenges in managing post-traumatic vestibular disorders and raising the risk of persisent symptoms and reduced quality of life. Data are not available on community or outpatient vestibular service provision, however literature relating to vestibular practice in the UK, Europe and Australia reveal a lack of education and training (38, 40, 43) which could be reflected in availability of resources.

### Preferred changes to practice

When asked about changes needed to improve the management of vestibular dysfunction, the overwhelming majority (97%) of respondents indicated that changes were necessary.

Key recommendations for improvement included the development of standardised protocols, more focused training and education, and the provision of additional resources such as case studies and instructional videos. Development of specific protocols or pathways may be difficult to formulate at the time of writing, due to the unavailability of specific cohort data in relation to effective treatment for post-traumatic vestibular dysfunction such as BPPV or vestibular hypofunction. Clinical practice guidelines do exist for assessment and treatment of idiopathic BPPV (44). However, direct application of these guidelines to the TBI population are problematic as these patients are more complex, often with co-existing limb and spinal injuries, and they may present with multi canal BPPV and experience more recurrences (45). Respondents’ suggestions reflect a desire for greater consistency in practice, as well as enhanced confidence and competence among healthcare professionals in managing vestibular disorders. Additionally, respondents noted that dizziness was often deprioritised in the acute phase of traumatic brain injury (TBI), with cognitive deficits being seen as more pressing. This has also been noted in other surveys of healthcare professionals managing patients with TBI (43). This highlights the need for greater recognition of the impact of vestibular dysfunction on patient outcomes and the importance of addressing dizziness in the acute care setting.

## Limitations

This survey was limited to all adult major trauma centres in the UK and Ireland but achieved a good response rate (87.5%). In most instances, one healthcare professional completed the survey on behalf of a team, and therefore some responses may reflect individual views or practice. This survey did not include healthcare professionals from trauma units not designated MTC status, or community rehabilitation units, both of which typically receive patients with a TBI in the sub-acute or chronic phases. Future work could extend this survey to these services to provide a more complete picture of service provision and evaluate whether clinicians experience of managing this cohort is associated with referral practices. Additionally, it is acknowledged that the survey was sent to healthcare professionals working in major trauma, and as a result may only reflect service provision for moderate severe patients with TBI, as patients with mild or symptomatic TBI (as per the Mayo criteria (2)) do not always attend hospital or may be discharged from Accident and Emergency without being admitted to a specialist acute trauma ward. Due to the majority of respondents being physiotherapists, it was not possible to explore associations between professional background and whether routine assessment or treatment were completed.

## Conclusion

The findings of this survey reveal several areas where post-traumatic vestibular dysfunction management can be improved across MTCs. Key challenges include geographic variability in practice, limited training, lack of standardised protocols, and inconsistent referral pathways. To address these issues, healthcare institutions should prioritise the development of formal protocols, increase training opportunities for healthcare professionals, and advocate for the inclusion of routine vestibular assessment and treatment. To facilitate this, high-quality randomised trials are needed to address gaps in evidence relating to treatment effectiveness. Furthermore, addressing geographic disparities and ensuring that patients have access to appropriate follow-up care are essential steps in improving outcomes, such as quality of life, for individuals with post-traumatic vestibular dysfunction.

## Data Availability

The raw data supporting the conclusions of this article will be made available by the authors on reasonable request.
