# Supplementary material for: Inconsistencies in care: a UK and Ireland survey exploring acute vestibular service provision in adult major trauma centres
Source: Front Neurol. 2026 May 25;17:1829958. doi: 10.3389/fneur.2026.1829958 (PMC13248885; doi:10.3389/fneur.2026.1829958)
Supplement: Supplementary file 1 [file Supplementary_file_1.DOCX]

Supplementary material

Supplementary material 1 – Copy of the survey

Survey of UK Major Trauma Centres care provision for patients with post-traumatic dizziness

This survey aims to evaluate current service provision in UK Major Trauma Centres for adults with dizziness following traumatic brain injury. We hope to understand what services are in place for this cohort of patients and whether any changes are needed to care pathways. This is a confidential survey and should take no more than 10 minutes to complete.

If you have any questions about the survey, please email Rebecca.Smith@imperial.ac.uk 

Please click on the arrow below to begin. Thank you for your time!

These questions are about you and where you work.

Question 1 At which Major Trauma Centre are you based?

________________________________________________________________

Question 2 What is your profession?

- Doctor
- Nurse
- Rehabilitation co-ordinator
- Physiotherapist
- Occupational therapist
- Other (please expand below) __________________________________________________

Question 3 How many years have you worked in trauma?  Please drag the slider to indicate how many years you have worked in trauma settings.

|  | Less than 1 years to 20+ years |
| --- | --- |

|  | 0 | 2 | 4 | 6 | 8 | 10 | 12 | 14 | 16 | 18 | 20 |
| --- | --- | --- | --- | --- | --- | --- | --- | --- | --- | --- | --- |

| How many years have you worked in trauma? | 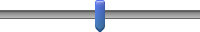 |
| --- | --- |

| Page Break |  |
| --- | --- |

The next few questions will ask specifically about you and your teams' awareness of, and training in, post-traumatic vestibular disorders.

Q4 Following traumatic brain injury, which vestibular disorders are you aware of that may be present and could be assessed for?

- Post-traumatic Benign Paroxysmal Positional Vertigo (BPPV)
- Post traumatic peripheral vestibular hypofunction
- Vestibular migraine
- Central vestibular dysfunction
- I am not aware of any of these
- Other (please expand below) __________________________________________________

Q47 How much training has your team had on post-traumatic vestibular disorders?

|  | Yes | No |
| --- | --- | --- |
| One or more members of the team has completed a masters (or above) level module or other qualification in vestibular practice |  |  |
| One or more members of the team has worked in vestibular rehabilitation or has completed a vestibular rotation |  |  |
| One or more members of the team have received in-house training on vestibular disorders |  |  |
| One or more members of the team have attended external courses on vestibular practice |  |  |

| Page Break |  |
| --- | --- |

Q48 These next few questions are about screening acute traumatic brain injury patients for vestibular conditions. In this survey, we have defined dizziness as a feeling of spinning, rocking, unsteadiness, imbalance, giddiness or similar.

Question 6 Are patients with post-traumatic dizziness assessed or treated on your major trauma ward?

- Yes, we assess and treat patients routinely
- We sometimes assess and treat patients
- No, we don't assess or treat patients

Display this question:

If Are patients with post-traumatic dizziness assessed or treated on your major trauma ward? = We sometimes assess and treat patients

Q55 If you assess patients with post-traumatic dizziness sometimes, in which instances do you assess?

- In patients who have subjective signs e.g. dizziness or lightheadedness
- In patients who have objective signs e.g.nystagmus, loss of balance during gait
- In patients who have a specific TBI feature (please expand below) __________________________________________________
- Other __________________________________________________

| Page Break |  |
| --- | --- |

Display this question:

If Are patients with post-traumatic dizziness assessed or treated on your major trauma ward? = Yes, we assess and treat patients routinely

Or Are patients with post-traumatic dizziness assessed or treated on your major trauma ward? = We sometimes assess and treat patients

Question 7 Who is currently responsible for **assessing and treating** traumatic brain injury patients with dizziness and/or imbalance? Please select as many options as appropriate.

- Trauma ward doctors
- Trauma ward physiotherapists
- Trauma ward occupational therapists
- Visiting specialist i.e. a neurologist or ENT doctor
- No one
- Other (please expand below) __________________________________________________

Display this question:

If Are patients with post-traumatic dizziness assessed or treated on your major trauma ward? = We sometimes assess and treat patients

Or Are patients with post-traumatic dizziness assessed or treated on your major trauma ward? = No, we don't assess or treat patients

Question 8 Why are patients with post-traumatic dizziness not routinely assessed or treated on the major trauma ward? Please select all that apply

- Staff time or capacity barriers
- Staff skill or knowledge barriers
- No protocol or pathway in place
- Patients are referred to outpatient services for further assessment and treatment
- Dizziness is a low clinical prority acutely
- No evidence to suggest need for acute assessment or treatment
- Other (please expand below) __________________________________________________

| Page Break |  |
| --- | --- |

Question 10 Do you have a pathway or protocol for assessing and treating patients with post-traumatic dizziness?

- Yes
- No

Display this question:

If Do you have a pathway or protocol for assessing and treating patients with post-traumatic dizziness? = No

Q56 If no, please tell us why. Please select all that apply

- No evidence for a pathway or protocol
- Insufficient knowledge or skills to develop or deliver one
- No outpatient vestibular rehabilitation service to refer to
- Lack of resources to deliver one i.e. staffing
- Other __________________________________________________

Display this question:

If Do you have a pathway or protocol for assessing and treating patients with post-traumatic dizziness? = Yes

Question 11 If yes, please upload your pathway or protocol. Alternatively please describe details of the protocol or pathway in the box below including details such as how patients are identified for assessment, who sees the patients, what is included in an assessment, who refers patients and where they are referred to.

Display this question:

If Do you have a pathway or protocol for assessing and treating patients with post-traumatic dizziness? = Yes

Q59 Please describe your protocol here

________________________________________________________________

________________________________________________________________

________________________________________________________________

________________________________________________________________

________________________________________________________________

| Page Break |  |
| --- | --- |

Display this question:

If Are patients with post-traumatic dizziness assessed or treated on your major trauma ward? = Yes, we assess and treat patients routinely

Or Are patients with post-traumatic dizziness assessed or treated on your major trauma ward? = We sometimes assess and treat patients

Question 12 Are patients assessed based on their **report of dizziness?**

- We assess patients ONLY if they report subjective dizziness or imbalance
- We assess patients REGARDLESS of whether they report dizziness or imbalance

| Page Break |  |
| --- | --- |

Display this question:

If Are patients with post-traumatic dizziness assessed or treated on your major trauma ward? = Yes, we assess and treat patients routinely

Or Are patients with post-traumatic dizziness assessed or treated on your major trauma ward? = We sometimes assess and treat patients

Q60 These next questions are about the content of your team's vestibular assessments.

Display this question:

If Are patients with post-traumatic dizziness assessed or treated on your major trauma ward? = Yes, we assess and treat patients routinely

Or Are patients with post-traumatic dizziness assessed or treated on your major trauma ward? = We sometimes assess and treat patients

Question 13 Which clinical components are included in a vestibular assessment? Please select all that apply

- Ocular motor (eye movement) examination
- Clinical Head thrust test
- Dix Hallpike test for posterior canal BPPV
- Supine head roll test for horizontal canal BPPV
- Lying - standing blood pressure
- Bedside test of static balance
- Bedside test of dynamic balance
- Test of hearing
- None of these
- Other (please expand) __________________________________________________

| Page Break |  |
| --- | --- |

These next questions relate to the assessment and treatment of post-traumatic benign paroxysmal positional vertigo or BPPV

Question 14 Does your team assess or treat *posterior* canal BPPV on the major trauma ward?

- Yes
- No
- I'm unsure what this is

Display this question:

If Does your team assess or treat posterior canal BPPV on the major trauma ward? = Yes

Question 15 In your team, **which clinicians and how many** are able to assess or treat posterior canal BPPV on the major trauma ward?

|  | None of these clinicians are able to assess or treat | 0-2 of these clinicians are able to assess and treat | 2-4 of these clinicians are able to assess and treat | More than 4 of these clinicians are able to assess and treat |
| --- | --- | --- | --- | --- |
| Physiotherapists |  |  |  |  |
| Occupational therapists |  |  |  |  |
| Doctors |  |  |  |  |
| Nurses |  |  |  |  |

Display this question:

If Does your team assess or treat posterior canal BPPV on the major trauma ward? = Yes

Question 16 How would your team treat posterior canal BPPV? Please select as many options as apply

- Epley manoeuvre
- Semont manoeuvre
- Log roll manoeuvre
- Brandt-Daroff exercises
- Advice
- I don't know

Display this question:

If Does your team assess or treat posterior canal BPPV on the major trauma ward? = No

Or Does your team assess or treat posterior canal BPPV on the major trauma ward? = I'm unsure what this is

Question 17 If your team doesn't treat posterior canal BPPV, please tell us why. Please select as many options as apply.

- Insufficient evidence to treat BPPV
- Not enough time or staff capacity
- Apprehensive about patients vomiting following treatment
- Apprehensive about completing treatment in this cohort
- Clinicians don't have skills or knowledge to treat
- Other __________________________________________________

| Page Break |  |
| --- | --- |

Display this question:

If Does your team assess or treat posterior canal BPPV on the major trauma ward? = Yes

Question 19 How **confident** is your team to assess and treat posterior canal BPPV?  Please move the slider to indicate confidence in assessment and treatment.

|  | Not very confident | Very confident |
| --- | --- | --- |

|  | 0 | 10 | 20 | 30 | 40 | 50 | 60 | 70 | 80 | 90 | 100 |
| --- | --- | --- | --- | --- | --- | --- | --- | --- | --- | --- | --- |

| Assessment of posterior canal BPPV | 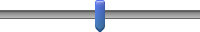 |
| --- | --- |
| Treatment of posterior canal BPPV | 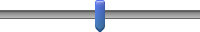 |

| Page Break |  |
| --- | --- |

Display this question:

If Does your team assess or treat posterior canal BPPV on the major trauma ward? = Yes

Q49 Are there aspects of posterior canal BPPV that your team is less confident with? Please select as many options as apply.

- Identifying which patients are appropriate to assess
- Completing the assessment
- Interpreting the eye movements
- Identifying the affected side
- Selecting the most appropriate treatment
- Establishing success or failure of treatment
- Differential diagnoses
- I am confident in all aspects of assessment and treatment of posterior canal BPPV
- Other __________________________________________________

| Page Break |  |
| --- | --- |

Question 20 Does your team assess or treat *horizontal* canal BPPV on the major trauma ward?

- Yes
- No
- I don't know what this is

Display this question:

If Does your team assess or treat horizontal canal BPPV on the major trauma ward? = Yes

Question 21 In your team, **which clinicians and how many** are able to assess or treat horizontal canal BPPV on the major trauma ward?

|  | None of these clinicians are able to assess or treat | 0-2 of these clinicians are able to assess and treat | 2-4 of these clinicians are able to assess and treat | More than 4 of these clinicians are able to assess and treat |
| --- | --- | --- | --- | --- |
| Physiotherapists |  |  |  |  |
| Occupational therapists |  |  |  |  |
| Doctors |  |  |  |  |
| Nurses |  |  |  |  |

Display this question:

If Does your team assess or treat horizontal canal BPPV on the major trauma ward? = Yes

Question 22 How would your team treat horizontal canal BPPV? Please select as many options as apply.

- Log roll manoeuvre
- Gufoni manoeuvre
- Li manoeuvre
- Forced side lying
- Advice
- I don't know

Display this question:

If Does your team assess or treat horizontal canal BPPV on the major trauma ward? = No

Question 23 If your team doesn't treat horizontal canal BPPV, please tell us why. Please select as many options as apply.

- Insufficient evidence to treat BPPV
- Not enough time or staff capacity
- Apprehensive about patients vomiting following treatment
- Apprehensive about completing treatment in this cohort
- Clinicians don't have skills or knowledge to treat
- Other __________________________________________________

| Page Break |  |
| --- | --- |

Display this question:

If Does your team assess or treat horizontal canal BPPV on the major trauma ward? = Yes

Question 24 How **confident is** your team to assess and treat horizontal canal BPPV?

|  | Not very confident | Very confident |
| --- | --- | --- |

|  | 0 | 10 | 20 | 30 | 40 | 50 | 60 | 70 | 80 | 90 | 100 |
| --- | --- | --- | --- | --- | --- | --- | --- | --- | --- | --- | --- |

| Assessment of horizontal canal BPPV | 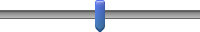 |
| --- | --- |
| Treatment of horizontal canal BPPV | 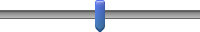 |

| Page Break |  |
| --- | --- |

Display this question:

If Does your team assess or treat horizontal canal BPPV on the major trauma ward? = Yes

Q50 Are there aspects of managing horizontal canal BPPV that your team are less confident with? Please select as many options as apply.

- Identifying which patients are appropriate to assess
- Completing the assessment
- Interpreting the eye movements
- Identifying the affected side
- Selecting the most appropriate treatment
- Establishing success or failure of treatment
- Differential diagnoses
- I am confident in all aspects of assessment and treatment of horizontal canal BPPV
- Other __________________________________________________

| Page Break |  |
| --- | --- |

Question 25 Does your team complete onward referrals for patients who have been diagnosed with BPPV?

- Yes
- No
- Sometimes

Display this question:

If Does your team complete onward referrals for patients who have been diagnosed with BPPV? = Yes

Or Does your team complete onward referrals for patients who have been diagnosed with BPPV? = Sometimes

Question 26 Where are patients referred to? Please select as many options as apply.

- Outpatient TBI clinic
- Outpatient vestibular rehabilitation
- Community rehabilitation
- GP
- In-patient specialist team
- Other __________________________________________________

Display this question:

If Does your team complete onward referrals for patients who have been diagnosed with BPPV? = Sometimes

Q45 If patients are referred on sometimes, please indicate the reasons for this. Select as many options as apply.

- Resources are borough specific
- Clinical indication - we don't refer routinely for all patients
- No pathway or protocol
- Other _________________________________________________

Display this question:

If Does your team complete onward referrals for patients who have been diagnosed with BPPV? = Yes

Or Does your team complete onward referrals for patients who have been diagnosed with BPPV? = Sometimes

Q44 Which patients are referred? Select as many as apply.

- Patients with suspected BPPV (i.e. not formally diagnosed)
- Patients who have been diagnosed with BPPV but not yet treated
- Patients who have had incomplete treatment i.e. treatment was not finished due to discharge
- Other __________________________________________________

Display this question:

If Does your team complete onward referrals for patients who have been diagnosed with BPPV? = No

Question 27 If BPPV patients are not referred, please tell us why? Select as many as apply.

- No clinical need to refer as patients have been treated already
- No pathway in place
- No community or outpatient specialists to refer to
- Other __________________________________________________

| Page Break |  |
| --- | --- |

Question 29 In your view, is assessment and treatment of post-traumatic BPPV **acutely** a low or high priority? Drag the slider to indicate whether it is low or high priority.

|  | Low priority | Medium level priority | High priority |
| --- | --- | --- | --- |

| Is BPPV assessment a low, medium or high priority in acute TBI? | 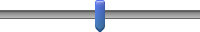 |
| --- | --- |
| Is BPPV treatment a low, medium or high priority in acute TBI? | 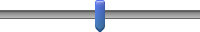 |

| Page Break |  |
| --- | --- |

Question 30 These next questions are about how your team manages patients with a diagnosed vestibular hypofunction (defined as a loss of peripheral vestibular function on one or both sides following a traumatic brain injury)

Question 31 Is someone in your team able to diagnose patients with a peripheral vestibular hypofunction ?

- Yes
- No
- I don't know what this is

Display this question:

If Is someone in your team able to diagnose patients with a peripheral vestibular hypofunction ? = Yes

Q54 Please indicate how patients would be diagnosed with a peripheral vestibular hypofunction. Select all that apply

- Abnormal clinical head thrust test
- Presence of a peripheral vestibular nystagmus
- As confirmed by vestibular testing i.e. a vestibular head impulse test, caloric test or similar
- Other __________________________________________________

Display this question:

If Is someone in your team able to diagnose patients with a peripheral vestibular hypofunction ? = No

Or Is someone in your team able to diagnose patients with a peripheral vestibular hypofunction ? = I don't know what this is

Question 33 If your team is currently not diagnosing patients with a peripheral vestibular hypofunction, please tell us why?

- Insufficient theoretical skills or knowledge
- Lack of practical skills
- Lack of confidence
- No access to vestibular function testing equipment i.e. vestibular head impulse testing or caloric testing
- No senior or consultant level support
- Unaware of the need to assess
- Other _________________________________________________

| Page Break |  |
| --- | --- |

Display this question:

If Is someone in your team able to diagnose patients with a peripheral vestibular hypofunction ? = Yes

Or Is someone in your team able to diagnose patients with a peripheral vestibular hypofunction ? = I don't know what this is

Question 32 Is acute treatment provided for patients with a diagnosed vestibular hypofunction?

- Yes
- No
- Unsure

Display this question:

If Is acute treatment provided for patients with a diagnosed vestibular hypofunction? = Yes

Or Is acute treatment provided for patients with a diagnosed vestibular hypofunction? = Unsure

Question 34 What treatments are provided for patients with a diagnosed vestibular hypofunction? Select as many as apply

- Advice e.g. maintaining 'normal' head movements
- Gaze stability exercises
- Static balance exercises
- Dynamic balance exercises
- Onward referral for outpatient vestibular rehabilitation
- Other __________________________________________________

Display this question:

If Is acute treatment provided for patients with a diagnosed vestibular hypofunction? = No

Q35 If patients are not provided with treatment for a diagnosed vestibular hypofunction, please tell us why?

- No evidence to provide treatment
- Insufficient time or staff capacity
- Unsure of correct treatment
- Patients are referred to outpatient vestibular rehabilitation services
- Other __________________________________________________

| Page Break |  |
| --- | --- |

Q51 Are patients with a diagnosed vestibular hypofunction referred on to other services?

- Yes
- Sometimes
- No

Display this question:

If Are patients with a diagnosed vestibular hypofunction referred on to other services? = Yes

Or Are patients with a diagnosed vestibular hypofunction referred on to other services? = Sometimes

Q52 If yes or sometimes, where are patients referred to? select as many as apply

- Outpatient vestibular therapy services
- Community neuro therapy services
- Consultant led neurology clinic
- Traumatic brain injury clinic
- Other __________________________________________________

Display this question:

If Are patients with a diagnosed vestibular hypofunction referred on to other services? = No

Or Are patients with a diagnosed vestibular hypofunction referred on to other services? = Sometimes

Q53 If no or sometimes, why are all patients not referred? Select as many as apply

- No community services to refer to
- No outpatient therapy or medic led clinic to refer to
- Borough specific resources
- Not always a clinical indication to refer
- Other __________________________________________________

| Page Break |  |
| --- | --- |

Q36 Do you think any changes are necessary to the way in which your trauma centre manages post-traumatic dizziness?

- Yes
- No

Display this question:

If Do you think any changes are necessary to the way in which your trauma centre manages post-trauma... = Yes

Q41 If so please tell us what sort of changes would help you?

- National guidelines
- Local protocol
- Better quality evidence relating to assessment and treatment
- Role models
- Supportive managers
- Training
- Other __________________________________________________

Display this question:

If Do you think any changes are necessary to the way in which your trauma centre manages post-trauma... = No

Q57 If not please tell us why

________________________________________________________________

| Page Break |  |
| --- | --- |

End of Block: Question Tour Block 3

Start of Block: Block 3

Please use this box to add any other details you think may be useful, otherwise click submit to finish the survey - thank you for your time!

________________________________________________________________

Supplementary material 2 – sites with two responses (n=6)

Sites 12 and 13 – Responses received from a Physiotherapist and Occupational therapist

| **Question** | **Yes** | **No** | **Interpretation** |
| --- | --- | --- | --- |
| Are patients with post-traumatic  dizziness assessed or treated ? | 2 - Sometimes | 0 | Yes – sometimes (practice confirmed) |
| Do you have a pathway or protocol in  place? | 0 | 2 | No (practice confirmed) |
| Are patients assessed based on their  report of dizziness? | 2 | 0 | Yes (practice confirmed) |
| Does your team assess and treat  posterior canal BPPV on the ward? | 0 | 2 | No (practice confirmed) |
| Does your team complete onwards  referrals for patients who have been  diagnosed with BPPV? | 1 | 1 | Practice unclear (conflicting response) |
| Does your team assess and treat  horizontal canal BPPV on the ward? | 0 | 2 | No (practice confirmed) |
| Can someone in your team diagnose a  vestibular hypofunction? | 0 | 2 | No (practice confirmed) |
| Are patients with a diagnosed  vestibular hypofunction referred on  to other services? | 0 | 2 | No (practice confirmed) |

Sites 16 and 17 – Responses received from two physiotherapists

| **Question** | **Yes** | **No** | **Interpretation** |
| --- | --- | --- | --- |
| Are patients with post-traumatic  dizziness assessed or treated ? | 1 - Sometimes | 1 | Practice unclear (conflicting response) |
| Do you have a pathway or protocol in  place? | 0 | 2 | No (practice confirmed) |
| Are patients assessed based on their  report of dizziness? | 1 | 0 | Yes (practice confirmed) |
| Does your team assess and treat  posterior canal BPPV on the ward? | 0 | 2 | No (practice confirmed) |
| Does your team complete onwards  referrals for patients who have been  diagnosed with BPPV? | 2-Sometimes | 0 | Yes (practice confirmed) |
| Does your team assess and treat  horizontal canal BPPV on the ward? | 0 | 2 | No (practice confirmed) |
| Can someone in your team diagnose a  vestibular hypofunction? | 0 | 2 | No (practice confirmed) |
| Are patients with a diagnosed  vestibular hypofunction referred on  to other services? | 1-Sometimes | 1 | Practice unclear (conflicting response) |

Sites 18 and 19 – Responses received from two physiotherapists

| **Question** | **Yes** | **No** | **Interpretation** |
| --- | --- | --- | --- |
| Are patients with post-traumatic  dizziness assessed or treated ? | 2 | 0 | Yes (practice confirmed) |
| Do you have a pathway or protocol in  place? | 2 | 0 | Yes (practice confirmed) |
| Are patients assessed based on their  report of dizziness? | 0 | 2 | No (practice confirmed) |
| Does your team assess and treat  posterior canal BPPV on the ward? | 2 | 0 | Yes (practice confirmed) |
| Does your team complete onwards  referrals for patients who have been  diagnosed with BPPV? | 2 | 0 | Yes (practice confirmed) |
| Does your team assess and treat  horizontal canal BPPV on the ward? | 2 | 0 | Yes (practice confirmed) |
| Can someone in your team diagnose a  vestibular hypofunction? | 2 | 0 | Yes (practice confirmed) |
| Are patients with a diagnosed  vestibular hypofunction referred on  to other services? | 2 | 0 | Yes (practice confirmed) |

Sites 21 and 22 – Responses received from two physiotherapists

| **Question** | **Yes** | **No** | **Interpretation** |
| --- | --- | --- | --- |
| Are patients with post-traumatic  dizziness assessed or treated ? | 2- sometimes | 0 | Yes (practice confirmed) |
| Do you have a pathway or protocol in  place? | 0 | 1 | No (practice confirmed) |
| Are patients assessed based on their  report of dizziness? | 1 | 0 | Yes (practice confirmed) |
| Does your team assess and treat  posterior canal BPPV on the ward? | 0 | 1 | No (practice confirmed) |
| Does your team complete onwards  referrals for patients who have been  diagnosed with BPPV? | 0 | 1 | No (practice confirmed) |
| Does your team assess and treat  horizontal canal BPPV on the ward? | 0 | 1 | No (practice confirmed) |
| Can someone in your team diagnose a  vestibular hypofunction? | 1 | 0 | Yes (practice confirmed) |
| Are patients with a diagnosed  vestibular hypofunction referred on  to other services? | 1 | 0 | Yes (practice confirmed) |

Sites 24 and 25 – Responses received from a nurse and a physiotherapist

| **Question** | **Yes** | **No** | **Interpretation** |
| --- | --- | --- | --- |
| Are patients with post-traumatic  dizziness assessed or treated ? | 2- sometimes and routinely | 0 | Yes (practice confirmed) |
| Do you have a pathway or protocol in  place? | 0 | 2 | No (practice confirmed) |
| Are patients assessed based on their  report of dizziness? | 2 | 0 | Yes (practice confirmed) |
| Does your team assess and treat  posterior canal BPPV on the ward? | 1 | 1 | Practice unclear (conflicting response) |
| Does your team complete onwards  referrals for patients who have been  diagnosed with BPPV? | 2-sometimes | 0 | Yes (practice confirmed) |
| Does your team assess and treat  horizontal canal BPPV on the ward? | 1 | 1 | Practice unclear (conflicting response) |
| Can someone in your team diagnose a  vestibular hypofunction? | 0 | 2 | No (practice confirmed) |
| Are patients with a diagnosed  vestibular hypofunction referred on  to other services? | 2-sometimes | 0 | Yes (practice confirmed) |

Sites 28 and 30 – Responses received from two physiotherapists

| **Question** | **Yes** | **No** | **Interpretation** |
| --- | --- | --- | --- |
| Are patients with post-traumatic  dizziness assessed or treated ? | 2 | 0 | Yes (practice confirmed) |
| Do you have a pathway or protocol in  place? | 1 | 1 | Practice unclear (conflicting response) |
| Are patients assessed based on their  report of dizziness? | 0 | 2 | No (practice confirmed) |
| Does your team assess and treat  posterior canal BPPV on the ward? | 2 | 0 | Yes (practice confirmed) |
| Does your team complete onwards  referrals for patients who have been  diagnosed with BPPV? | 2-sometimes | 0 | Yes (practice confirmed) |
| Does your team assess and treat  horizontal canal BPPV on the ward? | 2 | 0 | Yes (practice confirmed) |
| Can someone in your team diagnose a  vestibular hypofunction? | 2 | 0 | Yes (practice confirmed) |
| Are patients with a diagnosed  vestibular hypofunction referred on  to other services? | 2-sometimes | 0 | Yes (practice confirmed) |

Supplementary material 3

Table 1. Number of clinicians per centre able to assess and treat posterior canal BPPV

| **Healthcare professional** | **None of these clinicians** | **0-2 clinicians** | **2-4 clinicians** | **More than 4** |
| --- | --- | --- | --- | --- |
| Physiotherapists | 1 | 7 | 1 | 7 |
| Occupational therapists | 11 | 3 | 0 | 0 |
| Nurses | 11 | 3 | 0 | 0 |
| Doctors | 7 | 6 | 1 | 0 |
| **TOTAL** | **30** | **16** | **2** | **7** |

Table 2. Number of clinicians per centre able to assess and treat horizontal canal BPPV

| **Healthcare professional** | **None of these clinicians** | **0-2 clinicians** | **2-4 clinicians** | **More than 4** |
| --- | --- | --- | --- | --- |
| Physiotherapists | 0 | 5 | 4 | 3 |
| Occupational therapists | 9 | 3 | 0 | 0 |
| Nurses | 11 | 1 | 0 | 0 |
| Doctors | 8 | 4 | 0 | 0 |
| **TOTAL** | **28** | **13** | **4** | **3** |
